# Etiology of the Reigning Epidemic

**Published:** 1832-04-01

**Authors:** 


### ETIOLOGY OF THE REIGNING EPIDEMIC.

FACTS REGARDING THE REMOTE CAUSE OF THE EPIDEMIC CHOLERA.

1. *Orton's Theory of Cholera.*
2. *Topographical Facts on Cholera by the French Embassy in Russia.*
3. *Volta's Experiments on the Nature of Malaria.*
4. *O'Shaugnessy and Clanny's Analyses of the Blood in Cholera.*

---

### FACTS REGARDING THE REMOTE CAUSE OF THE EPIDEMIC CHOLERA.

HOWEVER obscure the nature of cholera may appear; or however unsettled may be its treatment; however divided we may be in sentiment upon the identity of the present fearful epidemic with the ordinary autumnal cholera that visits this country; and however the public mind may quail and tremble under the fear of its being possessed of a virulent communicative property by which it may propagate its seminal principles from house to house, and from country to country—we believe that, with a very few and unimportant exceptions, it is universally admitted that something either dissolved in the air, or emanating from the earth, is capable of producing it. We mean not either to deny or support the doctrine of its contagious character; nor shall we attempt to explain all the facts, nor reply to all the arguments which have been adduced in favour of or against that view. But it is our desire to lay before the reader in the succeeding pages a few remarkable and important facts in reference to the *principal remote cause* of the disease termed Asiatic Cholera, which, in our estimation, go far to show that this disease originated in a malarial principle, which, in all probability is nothing more or less than some gazeous fluid generated beneath the surface of the earth during the various processes of decomposition of vegetable substances.

After the thousand and one theories that have been started upon this subject, and have been consigned to annihilation, it may be hazardous to attempt any thing like novelty; but as we ground our opinion upon facts, and as these facts are so decisive as to lead, in our estimation, to one inference only, we hope to be excused for an opinion which the reader's own judgment may be voluntarily led to by a single perusal of the facts and statements upon which this opinion has been originally formed.

### 1. *Orton's Theory of Cholera.*

Great praise is due to Mr. Orton for the highly valuable collection of topographical and meteorological observations in regard to their influence on the manifestation of cholera in India. It is however surprising to see him taking into consideration for the remote cause of epidemics in general, and of cholera in particular, either the disordered state of the atmosphere, or sol-lunar influence; nay, that even of localities on the prevalence of the epidemic, and overlook or misinterpret so many important facts in support of an objectionable and quite fanciful hypothesis, which can never be either proved, or properly sustained, by philosophical reasoning.

He remarks, very properly, that as we do not see any particular set of functions peculiarly affected in cholera, we can not suppose that its proximate cause is to be ascribed to any particular organic lesion. The sudden defection of all powers of life requires no other cause but one of the most general agency throughout the frame to account for those circumstances. "It is an established position, (says he,) that the cold stage of intermittent fever is owing to diminished energy of the brain or nervous system; and in that affection we find nearly the whole of the principal symptoms of cholera, in a minor degree, and *scarcely any other*." From these views he concludes, that the primate cause of cholera consists in a diminution of the energy of the nervous system, which extends in various degrees to all the functions, and immediately produces the phenomena of the disease. On this hypothesis, he endeavours to explain all the symptoms of cholera, according to their manifestation in the different sets of functions of the human frame. This being accomplished, he comes to the investigation of the remote cause of epidemics in general. "Epidemics (says he) depend generally on some disordered state, or states of the atmosphere"—from which, it is evident, that the principal hopes of success in this inquiry into its nature must depend on ascertaining the meteorological occurrences which have accompanied the disease. A great many and valuable facts are related in the subsequent chapter, which proves that an extraordinary disorder in the state of the atmosphere has always preceded the epidemical appearance of cholera in India. From that disordered state of the atmosphere extraordinary rainy weather occurred, which originated numerous and extensive inundations from the overflowing of rivers. And so constant was this fact in connexion with the appearance of the epidemic cholera, that he concludes this chapter with the following remark. "I have not met with a *single instance* in which the disease is found, or stated to have appeared in the serene and settled weather which usually occupies so large a portion of the year in India, to such an extent as to be termed epidemical; surely its connexion with the opposite states of the atmosphere can not longer remain in the smallest degree doubtful. The observations on the state of the atmosphere in former appearances of this epidemic lead to the same conclusion. It appears, therefore, that of all the atmospherical phenomena, which have been mentioned as accompanying the disease, none are universally present, except those which indicate a diminution in the density of the air, and a *tendency to rain, and storms*."

Here Mr. Orton leaves us on the earth and flies up to the moon; and although in the following chapter he annouuces for our consideration the sol-lunar influence, he seems so much satisfied with that planet alone, that he entirely forgets the sun, as though in a tropical climate, like that of India, it had but little to do in favouring the processes of decomposition and evaporation which go on so actively in that country. We, however, do not intend to follow Mr. Orton's journey, leaving those who are curious to know the numerous facts which he has collected with great patience and care to peruse at their own leisure the long chapter in which they are detailed. For our part, we must say that, in general, we found nothing else but the argument of the "*post hoc, ergo propter hoc*" in all the facts therein adduced. However, Mr. Orton says, that "on perceiving the analogy between the cold stage of fever and cholera, *he* was led to suspect that the epidemic might be influenced by the moon; and on collecting all the dates of the appearance of the disease at particular places, which were then within *his* reach, *he* found that *nearly* the whole of them had occurred within the short period of the full or change." Connecting these facts with the others related in the former chapter, on the atmospherical vicissitudes, he draws at last this conclusion. It is evident then that these two series of observations on the circumstances attending the epidemic—"coalesce, and yield one result." On these data is founded the following axiom:—"The atmosphere during the prevalence of the epidemic is in a rarefied state, and exhibits a great tendency to part with its moisture, forming thick clouds, heavy rain, or haziness, and to become agitated by storms." "It remains to be considered how far these circumstances compose or are connected with the *great cause* of the disease."

This is the main object of the following chapter, in which the *primary cause of the epidemic* is taken into consideration. This chapter is worthy of the attentive consideration of the professional reader. "It is evident (Mr. Orton says) that any diminution in the density of the atmosphere must increase its capacity for electricity, and consequently diminish the quantity of that fluid which it possesses in a free state: and if it has been proved that a rarefaction of the air accompanies the prevalence of cholera, it follows that the disease is accompanied by a *diminution* of the *free electric fluid* in the atmosphere. It is this *deficiency*, produced in this and in other ways, which I consider the *great*, and *immediate cause of the epidemic*."

"Influenced (we may say of him what he states of Mr. Scott) too much by this prevailing opinion, which indolently shuts the door to all inquiry," he concludes this part of his work with the following leading principle—"The *deficiency* of *electric fluid* in the atmosphere produces a correspondent *deficiency* of that principle in the *arterial blood;* whence the nervous system is rendered incapable of separating and supplying a sufficient quantity of the '*nerveo-electric* fluid' to the wants of the animal economy; and that state which has been termed 'diminution of nervous energy,' with its infinite series of effects, is produced."

Pathologists usually divide the causes of disease into three different varieties: one is called *causa remota*, the other *causa occasionalis*, the third *causa proxima*. But in Mr. Orton's theory of cholera all these causes are so unlogically employed that one and the same cause is made at one and the same time the predisposing, remote, and immediate. His proximate cause is a *deficiency* of *electricity* in the arterial blood, by which a *deficiency* of the *nerveo-electric* fluid is occasioned: his remote cause is a *deficiency* of *electricity* in the atmosphere; and the rain, the rapid changes of temperature, the new and full moon, &c. are considered by him as the occasional causes of this disease, which produce a *deficient* state of *electricity*.

To this second edition of his Essay he adds a Supplement, which indeed contains a very complete and valuable collection of many interesting and unequivocal facts, and observations on the meteorological and topographical circumstances connected with the appearances of the disease in India. The subject of the sudden and rapid changes of temperature, the heavy falls of rain, the stormy weather, &c. are taken into consideration again, and in a more extensive manner. And although he is rather now and then puzzled in explaining some striking exceptions which go against his theory, he passes over them in the best way he can, contenting himself with the question—"In what then but the change in the electrical state of the atmosphere, which so constantly attends these occurrences, and precedes their development, can the deleterious element of the general change consist?" Moreover, there is in this Supplement an important section on localities in regard to their influence in producing the disease; and here we might have expected that Mr. Orton would have turned his eyes from the heavens, and looked downwards a little upon the earth, where by chance they might have been struck by the many low and damp grounds, covered by stagnant dirty waters, by the extensive rice-fields, the banks and bogs, encumbered with jungles; or his nose might have been assailed by the odoriferous effluvia arising from the putrefaction of vegetable matter; and this we might have the more reasonably expected, since he states that—"it is abundantly evident that all *malaria* countries have suffered in an especial degree from the epidemic; which further proves the identity of the great cause of fever with *one* of the causes of the epidemic cholera."

All this however is just for Mr. Orton as "dust in the balance;" for he informs us that "Read has found, that the *miasmata* from *vegetable putrefaction* occasion *negative electricity* in the air. Is not, then, the air of marshes (where the decomposition of such vegetable matter is continually going on, particularly in hot weather) negatively electric? And is not this an approach to the solution of that celebrated medical problem, the cause of the unhealthiness of marshes?" In this way these circumstances, which might have produced, at least, some modification of his former theory, are settled to his satisfaction.

It is only at the head of this Supplement that the subject of contagion is taken into consideration. Of this word something like a phantom is made, which wanders capriciously about, and now and then is making its appearance where there is no necessity for it, (at least as far as this disease is considered in India): at which we are not surprised, since Mr. Orton says, in another place, that " it has been *clearly shown* that that agent alone is *totally insufficient* to account for the prevalence of epidemics." However, as he does not tell us in what degree, or to what extent this *agent* operates, nor whether it stands on the positive or negative pole of his etiological battery, we are inclined to believe that this doctrine was introduced for the purpose of rendering his work more palatable to those medical gentlemen who might have been deterred from reading it, because contagion was excluded from his catalogue of causes.

Electricity is certainly one of the strongest powers in nature. But, as the steam-engine is to be considered the power by which all the complicated machinery to which it is attached are put in action; so when we speak of the atmosphere, we must consider, that it is nothing else but an aereo-elastic fluid which is more connected with the earth than with the heavens, nay, which constitutes an identical part with it. From the earth a great many gaseous fluids arise, which ascend more or less into the atmosphere, and mixing with it contribute in many ways to change, or alter its natural constitution, from which the infinite variety of meteorological phenomena arise.

The different appearances which accompany electricity, in its positive and negative characters, are not essential differences in the simple nature of that general principle; but are only modifications produced in its mode of manifestation from the different relations in which one body stands with reference to this agent towards another. It is not surprising, then, if Read found that the air at one end of a school-room, situated over a common-sewer, was negatively electric, whilst the open air, as well as that at the other end of the room, was in the opposite state. But we are also convinced that, if Mr. Orton had been seated over an electrical stool, and under the most positive influence of a powerful electric machine, his respiration would have been equally affected, if he had been obliged to inspire at some length the mephitic air arising from that common-sewer, " which gave an ill smell;" which, we dare say, was not at all comparable to the " resinous odour" of negative electricity.

What may be the effect on the organic constitution of an individual so exposed to the influence of malaria, we do not pretend to say; leaving for Mr. Orton to apply his electrical theory, if he likes, for the proximate cause of those diseases which could originate from it. But as far as respects their remote cause, we are quite convinced that it depends entirely on some deleterious principle which has been absorbed into the system, and which has altered the natural character of the blood. We have several times bled patients under similar circumstances, and we have always found a particularly dark fluid state of the blood; even when some process of inflammation had been produced in some organ, in consequence of some particular exciting circumstances, as of long laborious work under the hot rays of a vertical sun. In such cases this blood presented a very distinct buffy coat, but it was never cupped: on the contrary, it presented an elevated, convex surface, depressed in the margins, in consequence of the slight state of coagulation of the crassamentum, which might have been very easily reduced to a fluid consistence, if mixt up a little with the copious yellow-greenish serum, by which it was surrounded. Sometimes the buffy coat consisted of a slight grey milky pellicula, which was even not quite coagulated, but resembled a milk-greasy fluid covering the surface of the crassamentum. The sedative power of these miasmata was in some cases, and in some years so great, as to render bleeding, in many instances, impracticable, and never to be performed without serious consideration. We shall never forget the case of a lad, who had a quartan ague, for which many remedies had been tried without effect. Thinking that the disease depended on chronic inflammation of the liver, which was certainly very large, we thought it proper to bleed him. His simple fever rapidly changed into the most dangerous *pernicious choleric fever*, by which he would probably have lost his life, had we not had time enough, through its long periodicity, to give bark in proper quantity. We have practiced in a district of the dukedom of Parma, which is well known for the endemic intermittent fevers from malaria. They prevailed on the eastern side, where a low, a very rich valley exists, which had been dried up by intrenching a small river that joins the river Po, which runs on the other side westwardly. When the last part of the Autumn, or the beginning of the Winter, has been very rainy; or when, at the beginning of the Summer, the snow upon the mountains, where those rivers have their source, melts suddenly, they overflow their intrenchments; those low grounds are the most subject to be covered by water, which can only in part run away. Thus regular cultivation is prevented, and instead of bringing forth a regular productive crop, a luxuriant wild vegetation takes place, which principally consists of thick, spongy jungles, and other watery plants. Towards the commencement of the Autumn, a great quantity of water being evaporated, a slow process of decomposition takes place, which is formed by the remaining moisture, and by the powerful influence of the sun.

Towards evening a white heavy mist may be seen arising from those grounds, to some height above their surface, till with the advancing of the night it becomes less manifest or entirely disappears, when the air becomes suddenly chilly and very damp. This is the worst moment to be exposed to its influence. When the weather is very close this mist is thicker and rises higher, and its smell is stronger. After a heavy fall of rain, if the temperature becomes cool, and the weather clear and dry, (which is not always the case, as sometimes the temperature is more oppressive and suffocating after a fall of rain, and more particularly when the sky remains covered with a thin, interrupted *stratum* of white clouds,) the evaporation from the earth, the damp thick mist, and the bad smell cease entirely, or is only limited to the very spot where they originate. On the contrary, when the weather has been very dry through the Winter, when the Spring regularly increases in temperature towards the Summer, and of course when the melting of the snow has been going on slowly, these valleys remain dry and are cultivated, their power of vegetation is less luxuriant, and the jungles give place to a more useful kind of grass. Hence have we seen some years passing almost without any ague, or of a very simple, and more manageable character. The same circumstance can not be expected in the rice-fields which are always artificially overflowed by water at particular periods.

On the other side of the district near the Po, which we have just alluded to, is a dry sandy ground, covered close to the water by poplar woods. There ague does not exist, although the air is damp at night, and chilly. If any ponds are left on this ground in consequence of the overflow of the river, their water is always very clear and pure, and they never smell badly, because of the sandy character of the ground; proving that it is not the cool and chilly temperature, or the damp state of the atmosphere alone, which causes these intermittent fevers.

There are parts of Italy, and other hot countries in which intermittent fevers from malaria do not exist at all; and there often happen in the hottest part of the Summer such rapid changes of temperature in consequence of storms with heavy falls of rain, as those mentioned by Mr. Orton in India, yet no *malignant cholera* or intermittent fevers arise from them. Spasmodic complaints, indeed, may suddenly occur, nay, diarrhœa, or cholera if he likes; but how different are they both in their most important symptoms, result, and method of treatment? Every one who has lived in those countries must know by personal experience, that sleeping lightly covered in bed when a rapid change in the temperature of the atmosphere takes place in consequence of a sudden fall of rain, brings on very often severe cramps in the legs, or an attack of diarrhœa. A man slept in a field during a very hot night. He awoke in the morning with spasms of his limbs, which were followed by tetanus, from which he died. Very cold water drank when hot may produce colic, diarrhœa, or even cholera. These same complaints have been produced by stepping out of bed, and putting the naked feet upon the cold stones with which many apartments are paved in Italy. Such accidents as these may happen, and we do not pretend here to say from what cause; but who can say that these complaints are the same with the malignant cholera? So does it happen that cholera appears in certain seasons in this, or any other country, either in consequence of some rapid change in the temperature of the atmosphere, or from particularly unwholesome or indigestible food. Thus poisons, large doses of drastic purgatives, or sea-sickness, as Joseph P. Frank remarks, may produce cholera, but are these affections to be compared with the malignant cholera? The treatment by which they are generally cured is enough to indicate the difference. Opium, stimulants, warm-bath, magnesia, and other aperient medicines, blood-letting relieve the spasms, stop the diarrhœa, and vomiting; when these symptoms have subsided the disease is cured. In the malignant cholera, on the contrary, these symptoms may cease, but not the danger of the patient's life. He must very often run through another severe stage of the disease, which is the immediate consequence of the fatal effect of the sedative and deleterious principle existing in his system, and which poisons the very source of the vital power. Those atmospherical vicissitudes may have contributed very much in India to the production of those spasmodic complaints, in conjunction with fever from malaria, but without it they would have been of a quite different character. A great many instances, we dare say, were related as malignant, which were only simple spasmodic cholera. The following quotation from J. P. Frank's Epitome de Curandis Hom. Morb. (De Proflusis Part II. §. 674, de Cholera,) will be found very interesting. "Majoris in medicinæ artis exercitio momenti divisio est, choleræ in *apyreticam*, quæ sine febre incedit, et in *febrilem*, quæ *febris legitimæ*, *periodicæ*, et quidem *perniciosæ*, *plerumque tertianæ*, *symptoma* est, ac *loco paludosa, calido sub cœlo*, vel maxime infesta; cum prima, ubique frequentius hoc ipsa occurrat."

2. *Topographical Facts on Cholera by the French Embassy in Russia.**

In perusing a small pamphlet containing an official report from facts and observations on cholera morbus, collected by the French Embassy in Russia, we found, amongst other things of less importance, some topographical statements of very considerable curiosity.

"An important fact appears to be proved by the following observations: woods seem to diminish the choleric influence so much, that very woody districts situated between infected provinces have been entirely preserved from this destructive scourge."

"We have sent to Government a map of European Russia, where the progress and ravages of the cholera are marked by a dark ground, which is more or less dark according to the proportion of deaths compared with that of the inhabitants. There the disease, which is indicated by a black line, *(trait,)* may be seen making its entrance into Europe by Bakou and other points of the Caspian shores, and extending itself as clouds of smoke, more or less thick, which after having reached Tiflis and Astrakan, join together, then divide, and separating into different directions, go afterwards to ravage Russia, Austria, Poland, and Prussia. Between these clouds there are small spots left entirely white upon the map which denote the districts totally preserved from the cholera; and every thing seems to indicate that those privileged places principally owe their safety to the forests by which they are intersected and surrounded.

"But a still more decisive fact comes to sustain our opinion. Kristofsky island, situated in the middle of the populous islands of St. Petersburgh, and which communicates with them by two magnificent bridges, and, with the town by a thousand barges, which bring every day, and especially on Sundays, a great many people who go to take a walk in that charming place, Kristofsky island, we say, *has been completely preserved from the reach of the cholera*; there has not been a *single* patient in the three villages which it contains.

"And it is not to be supposed that the inhabitants of those villages were of a different nature from those of the town: all the abodes of this island are country-houses empty in Winter, and full of people in Summer, either noblemen, artists, or citizens of the town. During the cholera in St. Petersburgh, almost all the French players retired to Kristofsky, and not a single patient was found amongst them, while out of the small number of their companions who remained in town, many either died from the disease, or were seized with its most violent form.

"To what, then, may be ascribed the salubrity of Kristofsky, inhabited by the same people as St. Petersburgh, following the same regimen, and communicating with each other every day, if not to the influence of the superb forest which covers it? No other cause than the many neighbouring woods was found to explain its salubrity, for the island is a low and damp one, exposed every night to cold and heavy fogs, and fouled every Sunday by the excess of people who go there to gorge themselves with intoxicating liquors.

"We believe then that woods, and *probably the fir-tree more than any other*, have the property of destroying, of absorbing, or of neutralizing that *unknown cause, which generates the cholera, and which seizes the persons predisposed to it either by bad regimen or bad habits.*"

* Observations sur le Cholera-Morbus. Par l'Ambassade de France en Russie. Paris, October, 1831.

Similar observations have been made in Italy on the subject of intermittent fevers arising from the malaria of the marshy grounds. Those places most frequently subject to have agues are destitute of trees; when districts in their neighbourhood, not only *well cultivated*, but *planted with many trees*, are quite healthy. *Regular plantations* have been ordered by Government in some places, as Mantua for example, to improve the state of the atmosphere, and fever has since diminished in that place. It is also well known that the *selva sacra* was thought to preserve ancient Rome from the infectious influence of its bordering marshes ; and was held in veneration, because in former times its destruction brought on a malignant fever amongst its citizens. Facts of a similar kind have been also remarked by Dr. Rush in North America, not only as regards intermittents, but the yellow fever.

The following facts extracted from the German papers which have been lately published, as they bear upon this point, deserve attention.

" It is a remarkable circumstance that very few cases of cholera have occurred at Baden, a watering place about ten miles from Vienna, though the whole country round it has been suffering dreadfully from the epidemics. This exception is ascribed to the vapours constantly rising from the warm springs at Baden, which are strongly impregnated with sulphur, and unite in streams, which wander through the town. The same happy exception occurred to Baden at an earlier date, and on several occasions, when the plague was raging in and about the Austrian metropolis. It was the sulphuretted carbonic acid with which the atmosphere hanging over the promontory of Baku is saturated, which protected its inhabitants from the cholera, when every other quarter on that side of the Caspian was laid waste by its virulence two years ago."

### 3. *Volta's Experiments on the Nature of Malaria.**

These facts have recalled to our memory some curious inquiries made in Italy, in 1776, by the celebrated Volta, on the malaria arising from marshy grounds. As they may probably throw some light on this obscure subject, we consider them worthy of serious consideration.

Volta being informed that inflammable air was continually bubbling up from a particular spring, and thinking it might be produced by a slow subterranean process of decomposition of vegetable and animal remains, he imagined he could obtain the same result, where similar circumstances were present. With this preconceived idea he embraced the first opportunity of ascertaining it by facts.

Being in a boat on the Lago Maggiore, at Como, where he saw the water most muddy, he began to puddle under it with his stick, and perceiving that many bubbles of air arose instantly to the surface of the water, he collected it in bottles, and as soon as he reached home examined it. The smell alone induced him to predict to several friends, who were present, that this air would inflame if a light were approached to it. The experiment was tried, and it burnt with a tranquil bluish flame.

These inquiries were repeated in many other places, and neither pond, ditch, nor any other place where muddy and dirty water was to be found, escaped his observation. He always obtained more or less of the same result, (with one exception, where he found an air which in place of igniting instantly extinguished the flame) provided the ground underneath contained the smallest portion of vegetable substance. From a sandy and gravelled ground, over which water was flowing in a rapid stream he could not obtain any ; nor was any to be procured from the mud of the public streets, or roads.

These facts having been ascertained, he began to think that the presence of water might not be a condition necessary to obtain this result provided there was enough of vegetable and animal remains which had previously been moistened by it. He then examined some ground which had been left lately flooded in the neighbourhood of the same Lago Maggiore, digging up the ground in several places, and throwing water into it, and he found that the same inflammable air was generated as in his former experiments. Even in places where there appeared no water, but in which holes were bored in the earth with a stick, an air was extricated which was inflamed by a taper and burned in the same manner as the air collected in bottles in the other experiments. " It was amusing," says Volta, "in boring quickly through the ground in several places, to see,

* Volta's Works, Vol. III. Florence, 1816.

when the air was lighted, how the flame flew rapidly from one hole to the other, gushed out from them violently, and blazed furiously, more particularly if I pressed or stepped with my feet over the ground so as to squeeze out a greater quantity of air." These experiments were repeated in other places with the same result, and after he had decided upon their accuracy, he called it, " *aira nativa delle palludi*," native air of marshes. This air burns very slowly with a beautiful thick lambent flame, but it is necessary that the opening of the vessel in which it is contained be rather large, otherwise it will not burn so well, but with interrupted crackling noise, and with so very faint a light as scarcely to be perceived. If a taper is merely introduced into the vessel it will inflame the contained air ; but if it be thrust down towards the bottom of the vessel it is extinguished. To burn well it is necessary either to be exposed to, or mixed with a certain quantity of atmospherical air.

Volta gives a translation of a letter, dated Craven Street, April 10th, 1774, which Dr. Franklin wrote to Priestly, in which he gives an account of observations and experiments made in North America, on the very same subject. Over the surface of some rivers in New Jersey, and of a mill canal, Franklin understood that a flame had been excited which flickered, spread, and continued to burn for about half a minute when a light was approached to it. Franklin himself tried to obtain the same result here by puddling some stagnant water with a stick so as to force some bubbles of air to the surface of the water, with the view of lighting them with a taper. Although he could not succeed in this attempt, however, he says that he caught an intermittent fever, which he could not ascribe to any other cause, but to the putrid air which he inspired for a long time, while he was laying upon the ground to perform these experiments.

These facts being ascertained, Volta went afterwards to examine the burning grounds at Pietra Mala, on the top of the Appenines, on the road between Bologna and Florence; and at Velleia, on the mountain of the dukedom of Placentia. There he found that the flames covering those grounds were nothing else but the same air in combustion. He also collected it in bottles, by throwing over the burning places, a sufficient quantity of water to extinguish them, when air came bubbling copiously up to the surface of the water. He also bored the ground in several places, where flames did not exist, and flames arose from these holes when a taper was approached to them ; which grew higher when pressed or stepped over the ground with his feet. Many other interesting inquiries were made by him at the same time regarding the topographical condition of those places.

He afterwards discovered that the slightest electric spark was sufficient to inflame this air, even in close bottles, provided a proper quantity of atmospheric air was previously mixed with it. From this fact he explained the flying fires, and many other meteorological phenomena of a similar nature both in the earth and in the atmosphere, in calm and stormy weather, which happen in Summer in very hot climates, and are too commonly ascribed entirely to electricity. In like manner did Volta explain, and we think very properly, the extraordinary case which happened at those times, of a clergyman, whose name was Joseph Franchini, who threw out from his stomach some very fœtid gas which took fire when it approached the air. He ascribed it to the natural evolution of phosphuretted hydrogen gas into the stomach which takes fire as soon as it is exposed to the atmosphere. This clergyman had been suffering for a long time from dyspepsia. Connecting then together these, and many other similar facts concerning his inflammable air of marshy grounds, he ascended to the more majestic phenomenon of volcanos and earthquakes, which he considered to depend on the natural chemical decomposition of mineral substances produced by the continual action of sulphuric acid and water upon them within the earth, from which, as in a very small scale in our artificial preparations, hydrogen gas is obtained in very large quantity, which burst out in copious flame, and agitated the terrestrial globe.*

We will not at present return to Mr. Orton, nor enter into any discussion on the most probable cause of earthquakes, nor stop to consider whether it may be more in ac-

* It may be worth attention here to state that the new island which was lately discovered in the Mediterranean close to Sicily has disappeared and left no other trace of its former existence, than a vast whirlpool, in which the waters are agitated by constant eructations of subterraneous gases. It would be very interesting to endeavour to collect some quantity of them for chemical analysis.

cordance with well ascertained facts, to suppose that electricity, or the formation of *steam*, as Mr. Orton says, is the principal cause of that particular natural phenomenon. However, we do not conceive, why the escape of *steam* into the air should not produce or favour occasionally these epidemical visitations. At least, Mr. Orton would suppose that natural *steam* be just like that of our steam-engines, which is produced from the evaporation of pure water; had we to take, as something like a specimen of it, the copious vapours continually arising from volcanos, we dare say that they cannot be the most healthy effluvia to breathe.

After this imperfect, but we hope very convincing, account of Volta's experiments on the malaria of marshy grounds, one will be surprised to read the following passage from the Quarterly Journal, 1827, by Dr. Macculloch, with whose opinion Mr. Orton perfectly agrees, as appears from the following quotation, p. 419, Orton's.

"Experiments carefully conducted (as ought not to be doubted when Vauquelin has been engaged in them) have not detected even the presence of any new substance in the atmosphere of marshes, far less its nature. It is, however, *evident that it cannot be any of the hydro-carburetted*, or other chemical gases, which it has at different times been supposed; *while remaining thus in darkness*, the only test of its presence continues to be its effects as to disease which it produces on the human body."

When Volta was examining the burning grounds of Pietra Mala, and Velleia, he was informed that those natural fires were sometimes occasionally extinguished by violent blasts of wind, but that they increased very much by heavy falls of rain in stormy weather, and that a stream of water, and a lake near to those places, bubbled up a greater quantity of air, and had the appearance of boiling water although it was quite cold. Volta did not, like Mr. Orton, call for electricity to explain these facts, but thought that a greater quantity of his inflammable air could have been forced out during the absorption, or infiltration of water into the ground, or into some cavities under it, where a large condensed quantity of gas had been collected in consequence of a slow process of decomposition of some very large masses of vegetable and animal substances (as for example, a forest or a marsh) which had a long time ago been buried under a sudden cleaving of mountains or of the ground. This, which was a mere supposition when the high mountains of Pietra Mala were examined by him, became an incontestible fact when Velleia was examined, where it manifestly appeared that this old Roman town was buried by one of those extraordinary accidents which change so often the surface of our globe. Even in our days accidents of a similar nature have happened although on a small scale; but large enough to bury several houses in a situation distant from Velleia only about two miles. The burning spot is not very far from that buried town, in the lowest part of a declivity of the mountain, near a torrent. Volta remarks, that these burning grounds have been very erroneously considered of a volcanic nature, since there is not the least trace of any volcanic eruption. Earthquakes are to be felt more in these places than in others. The character of the earth in those burning grounds are such as to give every reason to suppose the pre-existence of large quantities of vegetable matters.—That this inflammable air did not originate in any bituminous matter contained within those portions of earth from which it was extricated he proved by analysis, which at the same time tend to shew that it maintains some connexion with chlorine and its compounds. He put six ounces of the earth of the burning ground of Velleia into a retort, and he subjected it to the intense action of heat. Although naturally black it changed into a grey colour, and shrunk into small hard masses. Amongst other products at the neck of the retort he found a substance resembling sublimed acid, and at the bottom a residual fluid which tasted strongly of muriatic acid. A similar analysis has been performed by Baron Diertich on the earth of Pietra Mala, and analogous products were obtained.

In exploring the particular vicissitudes which those natural fires undergo in dry and rainy weather, and having supposed that the infiltration of water could produce such an increased quantity of evolution of gas to increase them, the idea was suggested to Volta of a very simple apparatus by which he brought the thing to a degree of demonstration.

A large, long, square box of wood was properly filled with his inflammable air. Over the lid a great many holes were pierced, which were covered with a quantity of clay and mould laid upon its surface, which was so intermixt by a weed of grass as to imitate a natural meadow. Water was poured upon this artificial ground, out of a watering-pot, so as to resemble rain; when this water, filtering through the clay into the box, caused some of the contained air to ascend through the apertures in its lid to the surface of this artificial ground, and a taper was applied to it, it ignited just in the same way as at the firing spots of Pietra Mala, and Velleia. Over this ground he could perform all the other experiments, which were done there. Had the lid of his box been disposed in such a manner as to admit being pushed down into its cavity by pressure over its surface, we dare say, that even without water the gas would have escaped up through the holes, and increased the flames, just in the same way as when Volta pressed with his feet over the ground which he bored at Pietra Mala and Velleia.

These facts and observations might, in our opinion, explain more naturally, and in a less hypothetical way some of the anomalies which rather too often meet Mr. Orton in the many and very important facts which he has brought forward (more particularly in his supplement) and which are left sometimes entirely without any explanation at all, or are adapted to his electrical theories. If we consider the peculiar topographical nature of some of the Indian districts, which he gives us some beautiful and minute description of, we do not indeed see any reason why it should not happen that an increased evolution of marshy effluvia from great falls of rain, or by the march of large masses of people over those grounds ought not to take place, even though some other causes are not acting in combination with the former.

Marshy grounds, as those which we have seen so often in Italy, are composed of a very thick clay, very dark in colour, and soft or greasy to the touch when moist; but which becomes very hard, whitish, and brittle after very dry and hot weather. Their surface is covered with a hard crust, not very deep, underneath which the earth remains as black as almost jet, and gives out a particular smell, not always disagreeable, which is by some compared to that of chlorine. Such places very often contain quantities of worms, which find in it a great supply of nourishing substance.

We dare say that, if some people had to step, or walk over those grounds, a larger quantity of gas would rise up from the numerous cracks, and render the air more infectious; and if a light were brought to them perhaps this air might be ignited, as in the experiments of Volta. This supposition, which may appear at first rather fanciful, could, in our opinion, explain how certain portions only of travelling bodies, and more particularly their followers, have been especially exposed to the ravages of cholera, &c.

We are very much astonished to find Mr. Orton stating, that it is *a most striking and well-established fact* in the history of the disease in India, that troops are more subject to it in camp than in quarters, or when marching, or travelling; and afterwards to add, " but these facts give no idea of the dreadful extent to which this *simple, and apparently harmless circumstance* is capable of operating to the destruction of human life."

We are sorry that we cannot judge from our personal knowledge of the real topographical condition of the districts where those travelling bodies were marching. But were we to judge from some remarkable instances in which they have been described to us in Mr. Orton's book, we should say that the low, damp situations of marshy grounds; the rice fields, the thauk over which these travelling bodies either marched, or on which they were stationed, were sufficient causes for the origin of the disease; while the stormy weather and the rapid changes of temperature to which they had been exposed, were nothing else but simple exciting causes of the disease.

An important circumstance, entirely unnoticed in Mr. Orton's work, in regard to these travelling troops, has been related to us by Colonel Walker, who has been in service in India. These marching troops, not only sleep in the field quite exposed to the baneful effect of the malaria arising from the ground; but their marches take place *always at night*. Sometimes they are obliged to march along a small narrow passage through marshy ground, and forests, among an immense quantity of jungles and other marshy plants, which grow with rapidity, and with all the luxuriant vegetation proper of marshes in hot climates.

Is it then surprising if such troops, or any other travellers have been caught by the disease, when they were so exposed, and *in the best moment* to absorb the deleterious principle which produces it; when, on the other hand, they were in the mean time subject to the influence of its exciting causes?

Every one who has travelled in Italy must have experienced the bad effects of malaria in crossing the Pontine marshes; and more especially if they have travelled at night, in hot and close weather, in open carriage, lightly dressed, and particularly if they felt drowsy; no matter if the weather is fine or stormy, although in Winter the risk is a great deal less.

In August, 1814, when returning to our native country after an absence of six years, we travelled through the unhealthy valleys, from which malaria is originated, in an open carriage, and lightly dressed, in the first part of the evening. The night was very fine; the day had been very hot and dry; but we found the atmosphere damp and chilly, and the fields were covered with a white dense mist, not very high from the ground. A few days afterwards we were seized by a quartan ague. Of six persons of the travelling party we were the only one who suffered, and that probably in consequence of our long absence from that country. The inhabitants of malariæ countries all know the danger of sleeping with windows open, or of wandering about at night, in search of cool refreshing air.

Nor is it at all surprising if the disease has always shown in India a marked disposition to spread along the banks of the rivers, for which we do not want to have recourse to *human intercourse* for the explanation of this particularly remarkable circumstance. It is quite sufficient to direct our attention to the topographical description of the districts through which these rivers run. A single quotation from Mr. Orton's work may give some idea of what we state in regard to the southern division of the Indian peninsula. "The streams which cross that wide plain are continually diverted from their course on each side into canals and expanses of rice-ground, which attend them, and leave the sea, excepting in the rains, but a small tribute of their water." We dare say, on the contrary, that rivers in India are the proper places for malaria, and we need not wonder, if in these rivers, where many barges are going up or down, they should act in a larger scale in favouring the evolution of a larger quantity of gas, just as in a smaller way did Volta's stick when moved in the muddy waters of the *Lago Maggiore*.

The following interesting fact, just related to us by one of our fellow-countrymen, who was living in the same district where we had our birth, may be worth narrating, as its authenticity may be entirely depended on. This gentleman was travelling in a gig along a road which crossed a large plane of rice-field that stood on each side of it. It was during a fine night in July, and precisely in that time when the water covering the fields was allowed to flow out through two ditches, which where parallel, and near to each side of the road. In one part, where the road was lower, the water from the ditches had overflowed. Soon after he crossed this place, a column of a brilliant flame rose from the surface of the water on his right hand, and followed the course of his chariot for a long space; when, in ascending a high trench, and turning on his right, the wandering fire vanished when it reached and struck against the high bank of the trench. If he threw his whip towards it, the flame retired and soon followed him again. This phenomenon, which is not uncommon, tends to prove how easily a current of deleterious gases arising from stagnant waters or marshy grounds may follow corps of travelling troops, and more especially barges, or ships going up and down rivers, or sailing by sea, in which a kind of infectious atmosphere may exist. This mode of propagation of the infectious malady from one country to another has been so generally admitted, and every where distinctly observed, as to leave almost no ground to doubt of its reality; when, on the contrary, the supposition of its having been diffused by contagion meets everywhere with so many and striking exceptions, that it appears very often highly objectionable. From the facts which we have read in Mr. Orton's book alone, which are certainly very copious and valuable, we think no one can doubt that the principal and primary cause of the Indian malignant cholera is to be attributed to miasmata originating principally from the decomposition of the immense quantities of vegetable substances which are undergoing a process of decomposition under the surface of the earth, and which exhale a gas that has acquired a more virulent character by the extraordinary vicissitudes and natural phenomena which have obtained in that country for a progressive series of years.

Although we do not pretend to say that the very same principle has been the remote cause of the European cholera also, yet we may say that the facts known at this moment tend to support such a doctrine. On this subject, however, we shall decline speaking particularly until a greater number of facts has been laid before the public.

Had we now to ascribe the salubrity of Kristofsky Island, and several other woody districts in Russia, where the ground underneath is not marshy, and the vegetation is not so luxurious as in India, to the property which trees of a resinous nature, more particularly, have of absorbing carbon and hydrogen, of which elementary principles, we dare say, marsh miasmata chiefly consist; or if we would ascribe the salubrity of Baden, near Vienna, and the promontory on the Caspian shores, to the property of the sulphureous emanations of attracting hydrogen, and of facilitating the decomposition of the pestilential miasmata; the following questions would very probably arise against our supposition.

Why did this malaria spread over such a great extent of the world, and advance progressively in particular directions for so many years, and after reaching a place, leave it suddenly, to appear with the same violence in other localities? Why did not this disease appear before, where proper grounds existed for the evolution of similar miasmata?

Although we are quite aware of the difficulty or impossibility of giving a satisfactory answer to these queries, we must say, however, that a similar and almost identical disease was known and observed before; that it is even in our times known, no doubt, in a less severe degree, and certainly not so extensively as now, in the very same places where that malarious principle originates. Hence has it been described in Italy, in Spain, and even in London, by old authors; and in the two former countries diseases of a similar character are to be seen yet, with only this difference, that they are described and known under a less objectionable name, and less fear is attached to them. In the last December number of the French Medical Review, a paper on this subject has been inserted, which was written by Dr. F. Pauli, junior, of Landau, *(Rhénane Bavaria,)* in which he has pointed out the great resemblance, nay, identity of the malignant cholera, with the *pernicious intermittent*, and *remittent fevers*, which are not seldom seen in that malarious district, but which are now and then accompanied with *choleric symptoms.*

Had we not in August, 1829, a limited, although not less severe, manifestation of the very same malignant disease in a school at Clapham, in consequence of a very foul drain or cesspool having been opened, and its contents thrown out into a garden adjoining the school? In a day or two afterwards one of the boys was attacked; and in two days more, twenty others out of the total, thirty, were taken ill. Cases of a similar nature have been observed at many other periods and in many other places. Had not this malignant disease been confused with the common and simple spasmodic cholera, to which no other resemblance exists than in a particular set of symptoms, we could have obtained some more correct notion of its real pathological characters.

In explanation of the cause of the present extensive diffusion of this fatal disease, we wish to submit to the attention of our reader the following quotation from Gibbon's History of the Decline and Fall of the Roman Empire, (vol. vii. p. 418, § iii.) where at cap. xliii. he gives the history of the origin and progress of the great plague which ravaged the world in the time of Justinian. "Æthiopia and Egypt have been stigmatised in every age, as the original source and seminary of the plague. In a damp, hot, stagnating air, this African fever is generated from putrefaction of animal substances, and especially from the swarms of locusts, not less destructive to mankind in their death than in their lives. The fatal disease which depopulated the earth in the time of Justinian and his successors, first appeared in the neighbourhood of Pelusium, between the Serbonian bog and the eastern channel of the Nile. From thence, tracing as it were a double path, it spread to the East, over Syria, Persia, and the Indies, and penetrated to the West, along the coast of Africa, and over the continent of Europe.

"Such was the universal corruption of the air, that the pestilence which burst forth in the fifteenth year of Justinian was not checked or alleviated by any difference of the seasons. In time, its first malignity was abated and dispersed; the disease alternately languished and revived; but it was not till the end of a calamitous period of fifty-two years, that mankind recovered their health, or the air resumed its pure and salubrious quality."

At this period were presented very extraordinary natural phenomena; comets appeared, and earthquakes occurred several times each year in different parts of the world.

Now from analogy we would say, that India has been known as the birth-place of the malignant cholera. This extensive and very fertile country is intersected by numerous large rivers, which spread their waters over the low and equal plains to run slowly to the sea. This circumstance, favoured by the continual action of a vertical sun, produces an extensive progress of decomposition among the large quantities of vegetable substances, which are the result of the highly luxuriant vegetation of those productive countries.

By particular extraordinary natural phenomena, as excessive falls of rain, earthquakes, near approach of comets, &c. these deleterious effluvia are evolved, and emanate in larger quantities, and either by means of human intercourse, or by the influence of the natural currents of electricity, they are transported or diverge in certain particular directions, where proper matter for their attraction, or evolution is found. There they make sad ravages, and establish a temporary focus of infection.

Both plague and cholera owe perhaps to the different sources of the elementary principle from which their miasmata originate—the one chiefly from the animal, the other from the vegetable kingdoms—their characteristic quality, of the first being highly contagious, when the other is not naturally so, in the *true sense of the word*, but may manifest a certain contagious nature, when by contingent circumstances its natural constitution is altered, as when true typhus fever occurs in conjunction with cholera, and the like.—Hydrogen, carbon, azote, and oxygen are the elementary principles of which those classes of natural products are chiefly composed. Of these the last only is not possessed of a deleterious influence on the animal body: while the others either separately or in combination with each other, are highly obnoxious; and it is perhaps only in consequence of their combination with oxygen that their pernicious character is either lost, or highly modified in many instances. A superadded quantity of the former evolved from the earth, and diffused in the air, may induce in it an infectious property capable of producing the present, or any other malignant disease.

In a subject of such great obscurity as this, we hope to be excused if we attempt to throw some light over it, even by means of hypotheses, which being supported by facts, may give them a greater degree of probability.

### 4. *Drs. O'Shaugnessy and Clanny's Analyses of the Blood in Cholera.*

We are sorry to see that, since medical gentlemen have dedicated themselves to the analysis of the blood and other fluids evacuated by cholera patients, none of them have thought proper to direct their chemical inquiries to the air. And we are the more disappointed by their omission, since it was stated by Dr. Jækriken of Moscow, that the air of some cholera hospitals was discovered to be altered in its composition. Experiments of this matter might have produced some interesting result. As we mentioned the experiments instituted on the blood of cholera patients, we may here properly remark, that Dr. O'Shaugnessy obtained the following result:—

"1. The blood drawn in the worst cases of the cholera is unchanged in its anatomical or globular structure. 2. It has lost a large portion of its water; 1000 parts of cholera serum having but the average of 850 parts of water. 3. *It has lost also a great proportion of its natural saline ingredients.* 4. Of the free alkali contained in healthy serum, not a particle is present in some cases, and barely a trace in others. 5. Urea exists in the cases where suppression of urine has been a marked symptom. 6. *All the salts deficient in the blood, especially the alkali or carbonate of soda, are present in large quantities in the peculiar white dejected matters.*"

Such are the results of Dr. O'S.'s analysis, and they are indeed such as we should expect to find in a disease in which all the secretions are stopped, and in which all the lymphatic and lacteal vessels of the intestinal canal are pouring out, or, if we may say so, vomiting forth all the most fluid parts of the blood, which itself would probably have been ejected, as in some cases it was, if this inverted movement had not been so rapid, or the crassamentum of the blood not so soon condensated in the largest vessels.

Dr. Clanny of Sunderland instituted some analyses of the blood of two individuals who died of cholera. One was a man of the name of Elliot Todd, æt. 33. He was attacked on the 12th of December, 1831, at 2 o'clock, a.m. Blood was extracted by V.S. ad oz. vij. and half. He died at 7, p.m. of the same day. "This blood, on applying the tongue to it, had no taste, nor any particular smell: *he* also tasted it again, sometimes after it had been drawn. The colouring matter was tasted afterwards, the coagulated albumen, and the fibrin, but in them no taste was found, nor any smell. It contained no gases of any description: was black as tar.

The other individual was a sailor who was taken ill in October last. The blood of this man contained one cubic inch of carbonic acid in the sixteen ounces which were taken.

| | *The Sailor.* | *Elliot Todd.* |
|---|---|---|
| Water | 756 | 644 |
| Albumen, (coagulated) | 121 | 31 |
| Colouring matter | 59 | 253 |
| Free carbon | 32 | 66 |
| Fibrin pressed and dried | 18 | 6 |
| Muriates of soda and potassa, carbonate of soda, and animal extraction | 14 | 0 |
| | 1000 | 1000 |

The blood in Todd possesses only two ounces of serum, which was like serum of healthy blood in appearance."

If these results could be ascertained in many other cases they would be of the greatest importance to point out the real difference in the pathology of these two diseases, the common, and malignant cholera. They would tend to establish the important pathological principle laid down by Mr. H. Bell: that, in common cholera we have *depraved secretions*, producing great constitutional derangement: when in the malignant one there is *suspension* of *secretion*, resulting from a general disorder. In the first case the object of the physician is to *evacuate crudities*, and *correct disordered actions*. In the 2d the curative indications are to *relieve the symptoms*, and *to restore the function*.

About the particular advantages to be derived in the treatment of cholera from these experiments, we will abstain to say any thing now, as we expect to be in a short time favoured with a public report on this point. If, however, we were to judge from the nature of Dr. Clanny's celebrated discovery in the treatment of this disease—*a pyramidal cork*, and *a T. bandage!!!*—we should say that little indeed is to be expected.—From what has been stated by Dr. O'S. in the Westminster Medical Society, we should suppose that, from his experiments, he finds reason to advocate his proposed method of treatment in cholera, by injecting such salts into the veins as would restore to the blood its purple colour. On this subject we shall ask Dr. O'S. this question: if he had submitted some mineral substance to the decomposing power of a galvanic battery, and if the conductors of the battery were cut through, what could be the advantage of pouring additional quantities of acid into the troughs with the view of restoring the progress of decomposition?

N———.*

### XIII.

### REPORT OF THE MADRAS MEDICAL BOARD ON EPIDEMIC CHOLERA.†

(*Drawn up by Order of Government*, 1824.)

WHEN the Epidemic Cholera seemed to have completed its progress throughout the territories of this Presidency, it was intimated by Government to the Medical Board, that the interests of the medical science and of humanity, would probably be promoted by a history of that disease, as it had appeared amongst the population, as well as

* The above review has been drawn up by a distinguished foreigner, who has paid great attention to the subject, and is well conversant with etiology in general.—ED.

